# Peak, Fluctuation, or Mean? A Correlation Analysis of Long-term Intraocular Pressure Variation Parameters in Patients with Stable Glaucoma

**DOI:** 10.5005/jp-journals-10078-1240

**Published:** 2019

**Authors:** Ana Luiza B Scoralick, Carolina PB Gracitelli, Diego T Dias, Izabela Almeida, Michele Ushida, Syril Dorairaj, Fábio N Kanadani, Augusto Paranhos, Tiago S Prata

**Affiliations:** 1 Glaucoma Service, Department of Ophthalmology and Visual Science, Federal University of São Paulo, São Paulo, Brazil; Glaucoma Unit, Hospital Medicina dos Olhos, Osasco, Brazil; Department of Ophthalmology, Instituto de Olhos Ciências Médicas, Belo Horizonte, Brazil; 2,7 Glaucoma Service, Department of Ophthalmology and Visual Science, Federal University of São Paulo, São Paulo, Brazil; 3,4 Glaucoma Service, Department of Ophthalmology and Visual Science, Federal University of São Paulo, São Paulo, Brazil; Glaucoma Unit, Hospital Medicina dos Olhos, Osasco, Brazil; 5 Glaucoma Unit, Hospital Medicina dos Olhos, Osasco, Brazil; 6 Department of Ophthalmology, Mayo Clinic, Jacksonville, Florida, USA; 8 Department of Ophthalmology, Instituto de Olhos Ciências Médicas, Belo Horizonte, Brazil; 9 Glaucoma Service, Department of Ophthalmology and Visual Science, Federal University of São Paulo, São Paulo, Brazil; Glaucoma Unit, Hospital Medicina dos Olhos, Osasco, Brazil; Department of Ophthalmology, Mayo Clinic, Jacksonville, Florida, USA

**Keywords:** Intraocular pressure fluctuation, Intraocular pressure peak, Stable glaucoma

## Abstract

**Aim:**

To perform a correlation analysis between long-term intraocular pressure (IOP) variation parameters (mean, peak, and fluctuation) in patients with stable open-angle glaucoma (OAG).

**Materials and methods:**

A cross-sectional observational study was carried out, in which patients with stable OAG were consecutively enrolled. All patients had to have glaucomatous optic neuropathy and characteristic visual field (VF) defects. Key inclusion criteria were ≥5 VF tests, ≥3 disc photographs, and ≥3 years of follow-up without any changes in current medical regimen. Stable OAG was defined as nonprogressive VF results and absence of anatomical changes for at least 3 years. Long-term IOP variation parameters were obtained from isolated IOP measurements from each visit (minimum of five IOP measurements). The main outcome measure was the correlation between these IOP variation parameters.

**Results:**

Of the 63 patients studied, 37 (59%) were women, and the mean age was 61 ± 12 years. Among all the analyses, IOP mean and peak had the strongest correlation (*r* = 0.94; 95% CI = 0.92–0.97; *p* < 0.001). There were also significant correlations between IOP peak and IOP fluctuation (*r* = 0.84; 95% CI = 0.75–0.90; *p* < 0.001), and mean IOP and IOP fluctuation (*r* = 0.62; 95% CI = 0.43–0.75; *p* < 0.001).

**Conclusion:**

Most long-term IOP variation parameters evaluated seem to be highly correlated. Notably, the correlation between mean IOP and IOP peak was the strongest one. We believe this fact should be taken into consideration as their inclusion as individual variables in a multiple regression model could lead to misinterpretation of the data.

**Clinical significance:**

Different well-designed studies are conflicting regarding which long-term IOP variation parameter is more clinically relevant. Our findings suggest that collinearity issues could explain in part the discrepant results among these studies evaluating the relationship between long-term IOP variation parameters and glaucoma prognosis.

**How to cite this article:**

Scoralick ALB, Gracitelli CPB, *et al.* Peak, Fluctuation, or Mean? A Correlation Analysis of Long-term Intraocular Pressure Variation Parameters in Patients with Stable Glaucoma. J Curr Glaucoma Pract 2019;13(1):28–31.

## INTRODUCTION

Glaucoma is the leading cause of blindness in the world, but when detected and treated before its later stages, blindness is usually preventable.^1^ Until now, intraocular pressure (IOP) remains the only modifiable risk factor for glaucoma development and progression.^2^ Even though being a key parameter to evaluation glaucoma diagnosis and progression, IOP varies significantly, due to either factors associated with the measurement itself (such as the type of tonometer, examiner, fluorescein, and circadian cycle),^3^ to individual patient/ocular factors (such as central corneal thickness,^4^ corneal hysteresis,^4^ dehydration,^5^ glucose levels,^6^ and fasting).^5^

In this context, many studies have investigated IOP variation patterns and their relationship with glaucoma management.^7,8^ One of the main focuses of such studies was which IOP variation parameter would be the best predictor of glaucoma development or progression: mean, peak, or fluctuation. Surprisingly, contradictory findings have been reported, and there is still no consensus on this matter.^9,10^ We hypothesized that the lack of agreement between the above-mentioned study results could be attributed in part to the collinearity between all these different IOP variation parameters and the lack of standardization of the different tests. Therefore, in the present study, we performed a correlation analysis between long-term IOP variation parameters (mean, peak and fluctuation) in patients with stable glaucoma.

## MATERIALS AND METHODS

After institutional approval and informed consent had been obtained, we consecutively enrolled patients with stable open-angle glaucoma (OAG). All patients had to have glaucomatous optic neuropathy, associated characteristic VF defects, and open-angle on gonioscopy exam. Key inclusion criteria were ≥5 VF tests, ≥3 disc photographs, and ≥3 years without any changes in current medical regimen during the follow-up. Stable OAG was defined as nonprogressive VF results (both trend and event analyses) and absence of anatomical changes for at least 3 years. Eyes with incisional or laser glaucoma surgery, ocular trauma, uveitis, chronic use of steroids, and with any ocular disease (besides mild cataract) were excluded.

All VF tests were performed using 24-2 Swedish interactive threshold algorithm (Humphrey Field Analyzer II, Carl Zeiss Meditec, Dublin, USA), and they were excluded if presenting more than 33% fixation losses or false-negative errors, or more than 15% false-positive errors. VFs were reviewed and eliminated in the presence of artifacts such as lid or rim artifacts, fatigue effects, inattention, or inappropriate fixation. VFs were also reviewed for the presence of abnormalities that could indicate diseases other than glaucoma.

Long-term IOP variation parameters (mean, fluctuation, and peak) were obtained from isolated IOP measurements from each visit. A minimum of 5 IOP measurements were used for assessment of each long-term IOP variation parameter. Scatter plots were created and Pearson correlation (parametric correlations) coefficients between each parameter were calculated using MedCalc software (MedCalc Inc., Mariakerke, Belgium). The α level (type I error) was set at 0.05.

## RESULTS

**Figs 1A to C:**
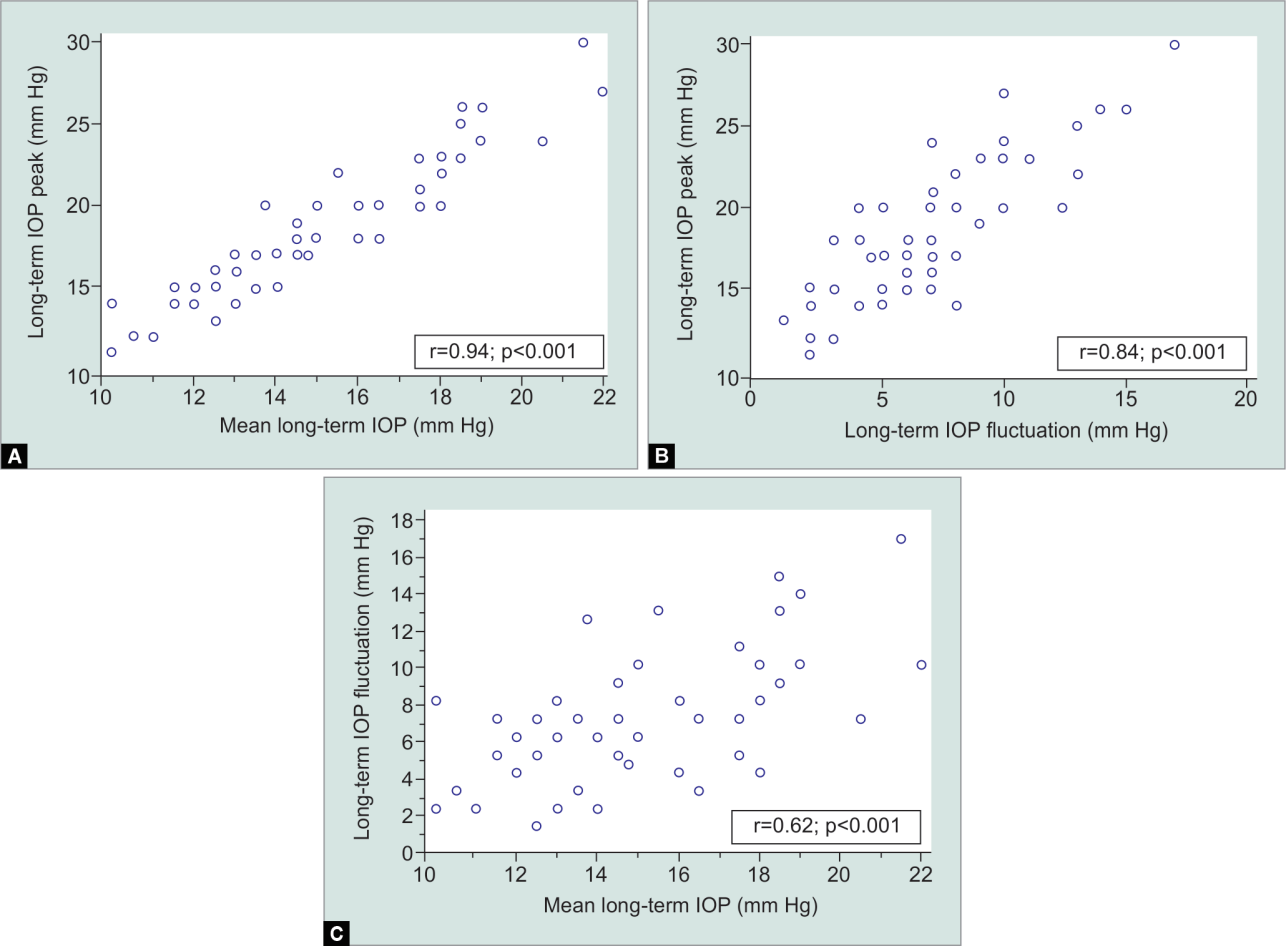
Scatter plot depicting the correlation between the (A) Mean IOP and IOP peak; (B) IOP peak and IOP fluctuation; (C) Mean IOP and IOP fluctuation

A total of 63 eyes (63 patients; mean age was 61 ± 12 years) were included. Demographic and clinical characteristics of study patients are provided in Table 1. Mean VF index was 92 ± 10%. Patients underwent a mean of 5.4 ± 2 VF tests, with a mean follow-up of 4.5 ± 1.3 years. There was a significant correlation between mean IOP and IOP peak (*r* = 0.94; 95% CI = 0.92–0.97; *p* < 0.001; Fig. 1A). Mean IOP peak also correlated significantly with IOP fluctuation (*r* = 0.84; 95% CI = 0.75–0.90; *p* < 0.001; Fig. 1B). Finally, there was a significant correlation between mean IOP and IOP fluctuation (*r* = 0.62; 95% CI = 0.43–0.75; *p* < 0.001; Fig. 1C). Among the three analyses we performed, IOP mean and peak had the strongest correlation.

## DISCUSSION

In the present study, we found significant correlations between all long-term IOP variation parameters in 63 consecutive stable OAG patients. Notably, the correlation between mean IOP and IOP peak was the strongest one, with a correlation coefficient of 0.94.

**Table 1:** Demographic and ocular characteristics of study patients

*Variables*	*Group (n = 63)*
Age (years)^*^	61 ± 12
Gender (F/M)	37 (59%)/26 (41%)
Race (C/O)	38 (60%)/25 (40%)
FU (years)^*^	4.5 ± 1.3
#VF^*^	5.4 ± 2.0
VFMD (dB)^*^	−3.7 ± 4.4
VFI (%)^*^	92 ± 11

F, female; M, male; C, Caucasian; O, others; FU, follow-up; #VF, number of visual fields during follow-up; VFMD, visual field mean deviation; VFI, visual field index

* Data are given as mean ± standard deviation

Looking at the conclusions from the World Glaucoma Association Consensus,^11^ one can note that there is no agreement on which would be the most adequate IOP parameter for glaucoma management. When analyzing the previously published data, even though there are several prospective robust studies that investigated long-term IOP variation parameters and their relationship with glaucoma prognosis, their findings are conflicting.^12,13^ For instance, several reports have found significant correlations between mean IOP values (but not IOP fluctuation) and glaucoma development^9,12^ or progression.^7^ On the contrary, some studies reported long-term IOP fluctuation as a more important factor for VF progression than mean IOP values.^10,13^ Finally, when it comes to IOP peak, there is some evidence suggesting that the maximum IOP value is a better predictor of glaucoma progression than mean IOP or IOP fluctuation.^14^

In this context, one might ask what are the reasons for these conflicting results. One plausible explanation seems to be the heterogeneous populations included in each study, as underscored by Caprioli and Varma.^15^ In an attempt to put all these data together, the authors suggested that patients with advanced damage with mean IOP values in the low teens may have a greater benefit from having lower IOP variations than those with early disease, controlled in the high teens. A second reason could be related to collinearity, which happens whenever two exposure variables are highly correlated. In our study, we documented a significant correlation between all long-term IOP variation parameters. The inclusion of highly correlated exposure variables in a regression model is not recommended, as it may lead to incorrect results and misinterpretation of the data. In this context, previous reports had already noted significant linear correlations between long-term IOP variation parameters (as secondary outcomes of these studies).^9,14^ Although a few studies have taken collinearity into consideration while performing their analyses, most of them have not.^9,12–14^ Therefore, we believe that collinearity issues may explain in part the discrepant findings between these studies investigating the relationship between long-term IOP variation parameters and glaucoma prognosis.

Some specific characteristics and limitations of our study should be considered. First, the correlations we found in this specific sample of stable OAG patients may not reflect the associations for other populations (e.g., eyes with prior surgical interventions, eyes with angle closure glaucoma). Second, although most included patients had initial or moderate glaucoma, some had advanced disease. As these patients often use multiple drugs and are more prone to adherence issues, one might expect a larger IOP fluctuation in these cases. This fact should be taken into consideration when analyzing patients with advanced glaucoma. Third, even though short-term IOP metrics can also be used to study IOP variation (diurnal IOP fluctuation, as assessed by diurnal tension curves or provocative tests), they were not investigated in the present study. However, as we are aware that the specific role of short-term IOP fluctuation in glaucoma management and prognosis still needs to be better elucidated, such investigation is being conducted in another ongoing study. Finally, although we discussed collinearity as a possible confounding factor to the relationship between long-term pressure parameters and glaucoma development and progression, the present study did not directly investigate this relationship.

## CONCLUSION

In conclusion, from a clinical perspective, all long-term IOP variation parameters seem to be complementary and relevant to glaucoma prognosis, but each contributing differently according to patients’ characteristics and disease severity. From a more scientific or investigative point of view, as all these long-term IOP variation parameters seem to be highly correlated, their inclusion as individual variables in a multiple regression model could lead to misinterpretation of the data. We believe this fact should be taken into consideration while analyzing the outcomes of studies evaluating the relationship between long-term IOP variation parameters and glaucoma prognosis.

## CLINICAL SIGNIFICANCE

Although IOP is the only modifiable risk factor for glaucoma, the ideal form of its evaluation is still not well established. As previously discussed, different well-designed studies are conflicting regarding which long-term IOP variation parameter is more clinically relevant. Our findings suggest that collinearity issues could explain in part such discrepant results.

## References

[1] Quigley HA,, Tielsch JM, (1996;). Rate of progression in open-angle glaucoma estimated from cross-sectional prevalence of visual field damage.. Am J Ophthalmol.

[2] Leske MC,, Heijl A, (2007;). Predictors of long-term progression in the early manifest glaucoma trial.. Ophthalmology.

[3] Sit AJ,, Nau CB, (2008;). Circadian variation of aqueous dynamics in young healthy adults.. Invest Ophthalmol Vis Sci.

[4] Kotecha A,, Crabb DP, (2009;). The relationship between diurnal variations in intraocular pressure measurements and central corneal thickness and corneal hysteresis.. Invest Ophthalmol Vis Sci.

[5] Oltulu R,, Satirtav G, (2016;). The effect of dehydration and fasting on corneal biomechanical properties and intraocular pressure.. Eye Contact Lens.

[6] Pimentel LG,, Gracitelli CP, (2015;). Association between Glucose levels and intraocular pressure: pre-and postprandial analysis in diabetic and nondiabetic patients.. J Ophthalmol.

[7] Heijl A,, Leske MC, (2002;). Reduction of intraocular pressure and glaucoma progression: results from the Early Manifest Glaucoma Trial.. Arch Ophthalmol.

[8] Nemesure B,, Honkanen R, (2007;). Incident open-angle glaucoma and intraocular pressure.. Ophthalmology.

[9] Bengtsson B,, Heijl A. (2005;). Diurnal IOP fluctuation: not an independent risk factor for glaucomatous visual field loss in high-risk ocular hypertension.. Graefes Arch Clin Exp Ophthalmol.

[10] Lichter PR,, Musch DC, (2001;). Interim clinical outcomes in the collaborative initial glaucoma treatment study comparing initial treatment randomized to medications or surgery.. Ophthalmology.

[11] Weinreb RN,, Garway-Heath DF, (2016.). Diagnosis of Primary Open Angle Glaucoma.

[12] Medeiros FA,, Weinreb RN, (2008;). Long-term intraocular pressure fluctuations and risk of conversion from ocular hypertension to glaucoma.. Ophthalmology.

[13] Caprioli J,, Coleman AL. (2008;). Intraocular pressure fluctuation a risk factor for visual field progression at low intraocular pressures in the advanced glaucoma intervention study.. Ophthalmology.

[14] De Moraes CG,, Juthani VJ, (2011;). Risk factors for visual field Progression in treated glaucoma.. Arch Ophthalmol.

[15] Caprioli J,, Varma R. (2011;). Intraocular pressure: modulation as treatment for glaucoma.. Am J Ophthalmol.

